# Editorial: Generative AI for brain imaging and brain network construction

**DOI:** 10.3389/fnins.2023.1279470

**Published:** 2023-09-05

**Authors:** Shu-Qiang Wang, Zhiguo Zhang, Fei He, Yong Hu

**Affiliations:** ^1^Shenzhen Institutes of Advanced Technology, Chinese Academy of Sciences, Shenzhen, China; ^2^Institute of Computing and Intelligence, Harbin Institute of Technology, Shenzhen, China; ^3^Centre for Computational Science and Mathematical Modelling, Coventry University, Coventry, United Kingdom; ^4^Department of Orthopaedics and Traumatology, The University of Hong Kong, Hong Kong, Hong Kong SAR, China

**Keywords:** generative artificial intelligence, brain data generation, brain network, brain-inspired computing, diffusion model

## 1. Introduction

Brain imaging plays an important role in exploring brain which is claimed to be the most complex thing in the universe. In recent years, there has been remarkable progress in the field of brain imaging techniques. Meanwhile, advancements in brain imaging technologies have greatly enhanced our understanding of brain networks. As an important tool for exploring the relationship between brain structure and function, brain network computation represents the most promising direction for artificial intelligence to achieve breakthroughs and advancements in the field of neuroscience. By employing artificial intelligence algorithms to integrate complementary features from multi-modal brain imaging, it is possible to uncover the connectivity characteristics of neural circuits and establish multi-level mapping brain network models based on function, structure, and organization.

Generative artificial intelligence (AI) has witnessed significant expansion, encompassing the utilization of available data to generate fresh content that exhibits comparable underlying patterns to real-world data. The fusion of these two realms, generative AI and neuroimaging, offers a promising path for delving into diverse domains of brain imaging and brain network computation, specifically in the realms of extracting spatio-temporal brain characteristics and reconstructing the topological connectivity of brain networks. Generative artificial intelligence assists researchers in learning and understanding brain functional mechanisms in a broader feature space under limited sample conditions. It aids researchers in designing efficient fusion methods capable of handling and correlating multimodal data and domain knowledge information. By integrating multimodal brain data with prior knowledge from neuroscience, it achieves complementary synergy between the cooperative semantic information and knowledge rules inherent in different levels and factors of the brain.

This Research Topic contains nine articles that can be broadly classified into three categories: (1) four articles primarily focus on the application of generative artificial intelligence methods for enhancing brain data, (2) three articles investigate the relationship between artificial intelligence and brain mechanisms to explore the functioning principles of the brain, (3) the remaining articles primarily address the application and development of artificial intelligence in the diagnosis of brain disorders.

Generative artificial intelligence is one of the rapidly evolving fields. It has gained significant attention due to its inherent advantages, especially in domains where data scarcity is a challenge. The current construction of functional brain connectivity networks from fMRI relies primarily on toolkits, leading to erroneous estimation of connection strength and suboptimal performance in disease analysis. Zuo et al. proposed a novel Adversarial Temporal-Spatial Aligned Transformer (ATAT) model, which realizes a region-guided feature learning network by taking the volume and location of anatomical brain regions into account. And it can adaptively adjust the boundary features of neighboring regions and capture the global functional connectivity patterns of distant brain regions. The validity and superior performance of the proposed model in early Alzheimer's Disease prediction and progression analysis were verified by conducting experiments on the ADNI dataset. Most artificial intelligence models used for neuroimaging classification tasks have limitations in their learning strategies and lack the ability for incremental learning. Cao et al. constructed the BNLoop-GAN model, which combines multi-modal brain networks and a multiple-loop learning algorithm to enhance the accuracy of prediction tasks for Alzheimer's Disease. This advancement tackles the limitations of current AI models employed in neuroimaging classification, which frequently suffer from the absence of incremental learning capabilities during batch training.

According to the study of Gong et al., this review summarizes the integration of advanced brain imaging techniques and generative AI models, which show promise for extracting spatiotemporal brain features and reconstructing topological connectivity of brain networks. It surveys four classic generative models and their applications in brain image computing and analysis. It also discusses challenges and future directions in utilizing these AI techniques for large-scale brain data analysis to understand brain structure-function relationships, aid diagnosis and treatment of brain diseases, and promote neuroscience research. However, issues like data privacy and individual differences pose challenges that need to be addressed through balancing data usage and model interpretability. There is still controversy surrounding the metrics and validation of synthetic images, and visual assessment can be a time-intensive task. Brémond-Martin et al. used similitude metrics and psychovisual evaluation to validate the quality of synthesized images. They compared the results obtained from these two evaluation methods and further tested the images in a segmentation task. The researchers discovered a correlation between certain metrics and psychovisual decisions, suggesting the potential of using specific combinations of blur metrics as possible alternatives to psychovisual evaluations.

Currently, artificial intelligence and medical imaging techniques advancements hold great significance for the progress of brain science and our understanding of the human brain. Jin et al. provided a comprehensive overview of computational methods for reconstructing optic nerve fibers from medical images in a review. It discusses the clinical importance of optic nerve fiber reconstruction for diagnosing neurological diseases and guiding neurosurgery. They describe two main reconstruction strategies, image segmentation and fiber tracking, with fiber tracking providing more detailed fiber structures. Both conventional and AI-based approaches are reviewed for each strategy, demonstrating the superior performance of AI-based methods. Xiong et al. designed an improved HHT-Microstate analysis method that combines the improved Hilbert Huang Transformation (HHT) decomposition with microstate analysis to investigate the differences in EEG microstate parameters in each frequency band in nicotine addicts. The method is effective in identifying substance addiction disorders and provides new ideas and insights for the brain research of nicotine addiction. Chen et al. reviewed the interplay between neuroscience and artificial intelligence, from AI's early inspiration from the brain to its evolution and remarkable performance with little neuroscience dependence. However, recent collaborations studying neurobiological explainability of artificial intelligence models reveal they may resemble biological computation despite no explicit neuroscientific modeling. The authors proposed a framework to evaluate brain-likeness of artificial intelligence models to enable further improvements under the intertwined development of both fields.

With the advancements in artificial intelligence technology and a deeper understanding of human brain mechanisms, artificial intelligence is playing an increasingly crucial role in the diagnosis of brain disorders. Wang et al. implemented a prior knowledge-based precise diagnosis of blend sign network from head computed tomography scans, which achieves accurate diagnosis of hybrid signs in head computed tomography scans by incorporating a priori knowledge and combining auxiliary tasks and self-knowledge distillation strategies. The method demonstrated superior performance in experiments and has the potential to assist physicians in reducing workload and improving efficiency in clinical practice. Zongren et al. came out with a multi-view brain tumor segmentation model based on cross-window and focal self-attention, which achieves excellent performance with high segmentation accuracy while limiting the computational cost by enlarging the receptive field by parallel cross windows and improve global dependence by using local fine-grained and global coarse-grained interactions, which provides a new solution for the field of brain tumor segmentation.

These articles cover a wide variety of topics including brain medical image enhancement, brain network generation and reconstruction, brain mechanisms exploration, brain-inspired computing, and brain disorders assisted diagnosis. These researches enrich the corresponding research fields with insightful methodologies and techniques, and ultimately offering alternative solutions to effectively enhance the robustness, generalization ability, and interpret ability for related tasks.

We hope that our readers will have a delightful experience when reading these excellent articles.

## Author contributions

S-QW: Conceptualization, Methodology, Writing—original draft, Writing—review and editing, Project administration. ZZ: Writing—review and editing. FH: Writing—review and editing. YH: Writing—review and editing.

